# Ventilator for the management of patients with severe pneumonia: A protocol of systematic review

**DOI:** 10.1097/MD.0000000000022386

**Published:** 2020-10-09

**Authors:** Jian-Rong Sun, Huan-Huan Wang, Long-Ze Zong, Wei-Wei Yuan, Zhi-Yuan Bai

**Affiliations:** a Department of Geriatric Respiratory Medicine, Cardiovascular and Cerebrovascular Hospital of Yan’an University Affiliated Hospital; b Department of Critical Neurology, Yan’an University Affiliated Hospital; c Department of Joint Surgery, Yan’an University Affiliated Hospital; d Department of Surgical Intensive Care Center, Yan’an University Affiliated Hospital, Yan’an, Shaanxi, China.

**Keywords:** efficacy, safety, severe pneumonia, ventilator

## Abstract

**Background:**

This study will assess the efficacy and safety of ventilator for the management of severe pneumonia (SP).

**Methods:**

This study will search the following electronic databases in MEDLINE, EMBASE, Web of Science, PsycINFO, Cochrane Library, CNKI, and Scopus from the beginning to present without language restrictions. Two authors will screen all records according to the eligibility criteria; assess study quality; and extract all essential data from eligible studies. If sufficient studies are included, we will pool the extracted data and carry out meta-analysis.

**Results:**

This study will summarize published studies to assess the efficacy and safety of ventilator for patients with SP.

**Conclusion:**

The results of this study may supply a genuine understanding of perspective from a scientific basis on ventilator for the management of patients with SP.

## 1 Introduction

Pneumonia is a common and serious disorder.^[1]^ Combined with influenza, it is a very frequent cause of infection-associated death around the world.^[2]^ It is reported that about 4 million adults suffer from this condition, and about 50,000 deaths annually in the USA.^[3,4]^ If it cannot be treated effectively and timely, it can progress to the severe pneumonia (SP).^[5–7]^ SP has higher morbidity and mortality, despite advanced treatment and critical care are applied to those patients.^[8–10]^

Ventilator is widely used to manage patients with SP.^[11,12]^ Although numerous clinical studies are reported to utilize ventilator for the treatment of SP, there are still inconsistent results.^[11–22]^ Thus, this systematic review will try to provide robust and powerful evidence to judge whether or not ventilator is effective and safe for the treatment of SP.

## 2 Methods and analysis

### 2.1 Study registration

We registered the present protocol on INPLASY202070052. We report it in accordance with the Preferred Reporting Items for Systematic Reviews and Meta-Analysis Protocol statement.^[23]^

### 2.2 Study eligibility criteria

Eligibility criteria are as follows:

1. All adult patients (aged more than 18 years old) who were diagnosed as SP will be included, regardless race, gender, and severity and duration of SP.
2. All potential randomized controlled trials that assessed the efficacy and safety of ventilator compared with other treatments in treating SP will be considered for inclusion, irrespective language, and publication status.
3. Outcomes include all-cause mortality, duration of hospital stay, duration of intensive care unit stay, secondary infections, and any expected or unexpected adverse event.
4. In addition, we will exclude any other studies, such as animal study, case report, case series, review, nonclinical study, uncontrolled trial, and quasi-randomized controlled trials.

### 2.3 Search strategy

This study will systematically search electronic databases in MEDLINE, EMBASE, Web of Science, PsycINFO, Cochrane Library, CNKI, and Scopus from the beginning to present without language restrictions. The search strategy with detailed terms of MEDLINE is presented in Table 1. We will modify identical search strategy for other electronic databases.

**Table 1:**
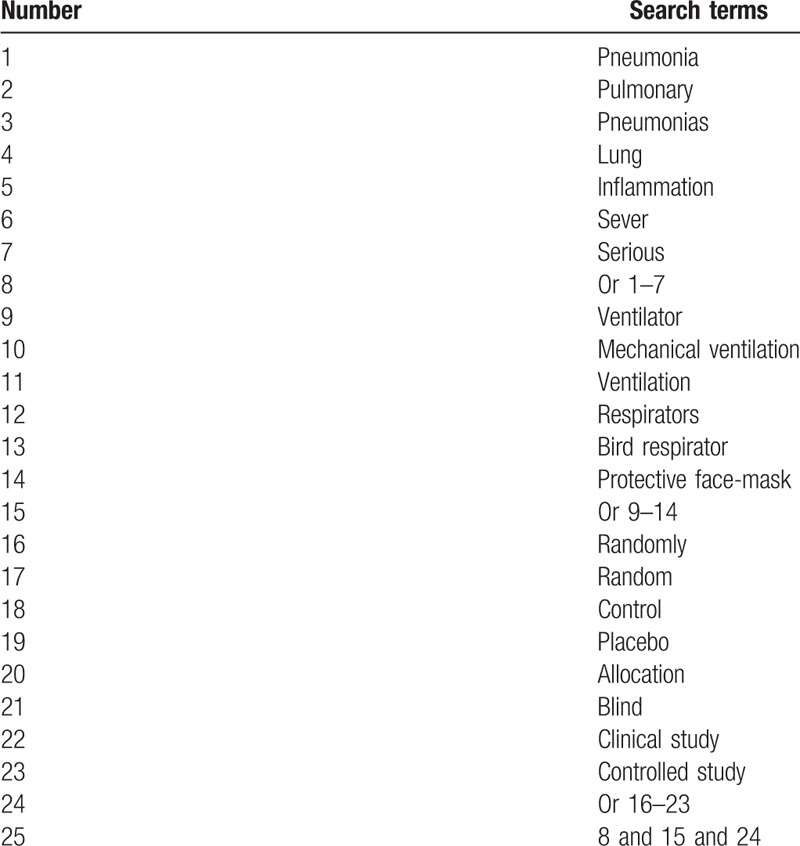
Detailed search strategy of MEDLINE.

Besides, we will search thesis, dissertations, conference abstracts, and reference lists of included studies.

### 2.4 Data collection and analysis

#### 2.4.1 Selection of studies

Two authors will independently screen titles/abstracts of included studies and will remove unrelated studies. After that, full papers of the remaining studies will be cautiously read against full eligible criteria. We will present the whole procedure of study selection in a flowchart. We will clarify any confusion with the help of a third author through discussion.

#### 2.4.2 Data extraction and management

Two authors will independently extract data according to the predesigned standardized data extraction form. Any dissimilarity between 2 authors will be solved by a third author via discussion. The extracted data includes title, first author, country, published year, patient information, sample size, study methods, details of modality, outcome indicators, safety, results, findings, funding information, and conflict of interest.

#### 2.4.3 Missing data dealing with

We will contact original trial authors to obtain any missing or unclear data by email. If we cannot obtain such data, we will analyze available data using intention-to-treat analysis.

### 2.5 Study quality assessment

Two authors will independently judge study quality using Cochrane risk of bias tool, which covers 7 aspects. Each item is further divided as high, unclear, and low risk of bias. Any incompatibility difference between 2 authors will be disentangled by a third author.

### 2.6 Statistical analysis

This study will perform statistical analysis using RevMan 5.3 software. We will estimate continuous data using weighted mean difference or standard mean difference and 95% confidence intervals, and will express dichotomous data using risk ratio and 95% confidence intervals. *I*^*2*^ test will be utilized to examine statistical heterogeneity across studies. It is interpreted as follows: *I*^*2*^ ≤ 50% means homogeneity, and we will place a fixed-effects model; *I*^*2*^ > 50% reveals considerable heterogeneity and we will employ a random-effects model. If homogeneity is identified and sufficient data are collected on the same outcome, we will plan to carry out a meta-analysis. Otherwise, we will find out possible sources of obvious heterogeneity.

### 2.7 Additional analysis

This study will carry out a subgroup analysis in accordance with the variations in study information, patient characteristics, study methods, and study quality.

This study will perform a sensitivity analysis to test the stability of study findings by removing low-quality studies.

This study will explore reporting bias using funnel plot and Egger regression test if more than 10 trials are included.^[24,25]^

### 2.8 Ethics and dissemination

This study does not need ethical documents, since no individual patient data will be obtained. We plan to publish this study at a peer-reviewed journal.

## 3 Discussion

Studies suggest that ventilator benefits for patients with SP; however, the evidence from previous clinical trials is inconsistent.^[11–22]^ In addition, we do not identify insufficient evidence-based medical evidence addressing this issue. With an increasing number of clinical studies, this proposed systematic review aims to appraise the efficacy and safety of ventilator for the treatment of patients with SP. It will summarize the up-to-date evidence of ventilator for SP. The findings of this study will provide evidence to determine whether ventilator is effective and safe for patients with SP, which may benefit patients, clinicians, and future researches.

## Author contributions

**Conceptualization:** Jian-Rong Sun, Huan-Huan Wang, Long-Ze Zong, Zhi-Yuan Bai.

**Data curation:** Jian-Rong Sun, Long-Ze Zong, Wei-Wei Yuan.

**Formal analysis:** Huan-Huan Wang, Long-Ze Zong, Wei-Wei Yuan, Zhi-Yuan Bai.

**Investigation:** Wei-Wei Yuan.

**Methodology:** Huan-Huan Wang, Long-Ze Zong, Zhi-Yuan Bai.

**Project administration:** Wei-Wei Yuan.

**Resources:** Jian-Rong Sun, Huan-Huan Wang, Long-Ze Zong, Zhi-Yuan Bai.

**Software:** Jian-Rong Sun, Huan-Huan Wang, Long-Ze Zong, Zhi-Yuan Bai.

**Supervision:** Wei-Wei Yuan.

**Validation:** Jian-Rong Sun, Huan-Huan Wang, Wei-Wei Yuan, Zhi-Yuan Bai.

**Visualization:** Jian-Rong Sun, Long-Ze Zong, Wei-Wei Yuan.

**Writing – original draft:** Jian-Rong Sun, Long-Ze Zong, Wei-Wei Yuan, Zhi-Yuan Bai.

**Writing – review & editing:** Jian-Rong Sun, Huan-Huan Wang, Wei-Wei Yuan, Zhi-Yuan Bai.

## Abbreviations

Abbreviation: SP = severe pneumonia.
